# Supplementary material for: Designing a brief and simple intervention to help young people with type 1 diabetes to live well: Protocol for developing a novel intervention with participation from young people
Source: PLoS One. 2023 Sep 8;18(9):e0285300. doi: 10.1371/journal.pone.0285300 (PMC10490951; doi:10.1371/journal.pone.0285300)
Supplement: S1 Appendix — (PDF) [file pone.0285300.s001.pdf]

Appendix S1: Interview Guides

Protocol 2022-9871

University of Brighton

University of Brighton protocol 2022-9871
For use in this study only

Page 2 - 1:1 research interview with Young Persons with Type 1 Diabetes (part 1 of study)
Page 11 - 1:1 research interview with SIBLINGS/PEERS of Young Persons with Type 1 Diabetes (part 1 of study)
Page 18 - Facilitator Guide for Focus Group Discussion with YPT1D (part 2 of study)

Interview guide #1

1:1 research interview with Young Persons with Type 1 Diabetes (part 1 of study) This checklist is a reminder to the researcher to cover specific areas within the interview. Below the checklist is the interview guide that will be used to guide the conversation

CHECKLIST	
Introduction	
Introduce self by name	
Qualify what name they would like to be used in the interview	Name (first name/nickname only):
Confirm they have had the participant information sheet – share / read through if not	
Advise on anticipated duration of call	
Confirm agreement to recording the interview and explain data handling	
Advise not to use people's full names. Suggest they use terms like "my doctor" or "my teacher" or initials instead of stating their name	
Explain the topics of the discussion today	
Explain what the information will be used towards	
Ensure informed consent is captured	
Discussion	
Cover each topic as indicated below	Note here any topics not covered, and reason why:
• Setting the scene	
• Diabetes and life	
• Diabetes and family	
• Diabetes and friends/school/work/significant other	
• Diabetes and healthcare team	
• Diabetes in general	
Interview close	
Check for any outstanding comments or reflections	
Thank the participant	
Ask if they have a brother/sister/friend who we could speak to as well. Explain why	
Explain reimbursement and confirm details	
Confirm if they would like to receive the final report about the research once we have developed and tested the intervention.<br>Where would they like this sent to?	
Ask if they would like to participate in a focus group	

University of Brighton protocol 2022-9871
use in this study only

Introduction

Information about this section (NOT for discussion with participant):

This section is to build rapport, ensure the participant understands about the research interview, confidentiality and consents to participating.

Thank you for meeting with me today to talk about your experiences of living with diabetes.

My name is [insert interviewer name]. What would you like me to call you as we chat? Please just use a first name or a nickname.

Can I just check that you got the participant information sheet from me or your nurse?

Before we start, let me explain what our call is about today. I'm a researcher, here to learn about how diabetes fits into your life. I'm keen to learn what are the most difficult things about having diabetes and what is important to you right now. What you share will help us to design a toolkit that seeks to help people in their late teens/early twenties to build their lives around diabetes.

As we discuss this, I would welcome your honest thoughts and feedback – not what you think I might want to hear. There are no right or wrong answers. I'm not here to tell you what you should/shouldn't be doing or how you should/shouldn't be feeling. It's your personal story that I'm interested in.

Everything you say is completely private. That's why we are just going to use first names – it means that no-one else will know that it was you who said any of the things you tell me. The only time I would need to speak to someone else is if I was concerned for your (or another person's) immediate safety. I would ask you if that's ok and you could tell me who you would prefer me to speak to.

University of Brighton protocol [illegible]-9871
For use in this study only

Our chat will last no more than 1 hour. If you need a break, or a snack, at any point please just say. We can easily take a break if needed.

When we set this meeting up, you said you were happy to do the call on your own. I just want to check that's still ok as you can have someone with you if you prefer.

Are you happy to continue with this research talk today?

- [If timing is an issue – agree appropriate next steps with participant eg rescheduling to a more convenient time]

Before we start I need to make sure that you understand what we are using the information you give me for and that you are agreeing freely to participate. This is called "informed consent". Have you had the informed consent sheet?

- [If so – check you have evidence, and reconfirm they are happy to proceed.

  - "Thanks for completing the form for me. Can I just confirm you are still happy to proceed today?"]
- [If not – read through Informed Consent sheet and ensure verbal agreement to proceed. Send IC form via email afterwards]
  - "No problem. I do need to make sure I have your informed consent though so let me read through the points with you and you can tell me if you agree with each of them. I will send you the form afterwards for your records."

- [If informed consent is not obtained, do not proceed]
  - "That's absolutely fine. You are free to decide whether or not to take part and I respect your decision not to participate. If you change your mind in the future, please feel free to send me a message but for now I understand that you don't want to participate. I wish you all the best for the rest of the day."

If you agree, I would like to record our talk so I can make sure to write good notes and really understand your experiences. The only people who will listen to the recording are me, and two other researchers in my team.

Are you happy for me to record our conversation?

- [If yes – start recording]
- [If no - check what time they have available. Explain 'we will likely need a little more time for our chat. Is that ok today? It's just that I need to write notes as we talk.]

State interview number and date for the recording

University of Brighton protocol 2022-9871
For use in this study only

Diabetes discussion

Section one – setting the scene	
Information about this section (NOT for discussion with participant):<br><br>This section is to build further rapport, and give the participant a chance to open up and start talking. Day to day management will be used as a mapping question linked to age and duration of T1D	
Please tell me a little bit about yourself? How old are you, who you live with, what you do, etc?	5 mins
How long have you had diabetes?<br>- When were you diagnosed (month-year if possible or age)?	
How do you manage your diabetes day to day (eg blood sugar monitoring, insulin, food, etc)	

Section two (2.1)– diabetes and life	
Information about this section (NOT for discussion with participant):<br><br>This section is to understand general insights and challenges about living with diabetes. In particular, listen for perceived limitations or difficulties	
What is it like living with diabetes day to day?<br>- What, if anything makes it hard to live with diabetes?<br>- What words would you use to describe diabetes?	10 mins
Sometimes there are specific times in life that really stick in our minds. Are there any specific examples you remember where diabetes made it hard for you to do something?<br>- Tell me about that.<br><br>Alternative Q (use if they struggle with above Q): What has been the hardest thing in the last week about having diabetes?<br>- Could anything have make it easier or more difficult	
How much do you think about diabetes day to day?<br>- Tell me about those thoughts?<br>- (If needed, can say "For example things like "what's my blood sugar now" or "I want to eat but it's difficult to do my insulin right now" or "oh no, I should have had a snack before playing football" or "why does this happen to me")	
Where do you get information about living with diabetes?<br>- Do you look online (social media sites/apps, etc). If so, what sites/groups do you use?	
What advice would you give to someone else with diabetes about how to deal with diabetes day to day?	

University of Brighton protocol 2022-9871
For use in this study only

Section two (2.2) – diabetes and family	
Information about this section (NOT for discussion with participant):<br><br>This section is to understand insights and challenges about family life for a diabetic. In particular, listen for causes of family conflict and causes of any relationship difficulties.	
How does diabetes fit into your family life?<br>- (If needed, can say "Do your family help at all, or they tell you what they think you should do?)<br>- (If needed, can say "Have there been times when diabetes has caused difficulties or arguments with any family members?<br>o Tell me about that.	10 mins

○ What do you wish you could have done differently in that example?")	
Roughly how old were you when you took on full management of your diabetes?<br>- Who decided that?<br>- How did you feel?	
Is anyone in your family very good at dealing with your diabetes?<br>- If so, who and why?	
Is anyone in your family not good at dealing with your diabetes?<br>- If so, who and why?	
What advice would you give to someone else with diabetes who was having difficulty with family members regarding their diabetes?<br>- If needed, can say "Would you recommend any specific words or actions that might help them?"	
How would you cope with your diabetes if you family weren't there for a week?<br>- How would you feel about that?	
FOR INDIVIDUALS WHO HAVE MOVED OUT OF THE CHILDHOOD HOME:<br>What was the hardest thing about moving out of home?<br>What you recommend any specific words or actions that might help other people with diabetes who are about to leave home?	

University of Brighton protocol 2022-0871
For use in this study only

Section two (2.3) – diabetes and school/college/university/work and friends/significant other	
**Information about this section (NOT for discussion with participant):**<br><br>This section is to understand relationships with peers and/or partners. In particular listen for issues around disclosure, comments from peers, avoidance strategies to managing diabetes.	
Tell me about how diabetes fits into school/college/university/work?<br>- Are there any times when it's hard to deal with your diabetes at school/college/university/work?	10mins
What would be the best way to deal with diabetes at school/college/university/work?<br>- Is there anything that stops you doing that?	
Do you tell your friends/significant other that you have diabetes?<br>- If yes: How easy or difficult was that? Tell me about it?<br>- If no: What holds you back from telling them? How do you think you would feel if they knew you had diabetes?	
What, if anything, do your friends/colleagues say about diabetes?<br>If yes: How does that help you, if at all?<br>If no: Would you like them to say anything or help you ever? If so, how?	

Section two (2.4) – diabetes and healthcare team	
Information about this section (NOT for discussion with participant):<br><br>This section is to understand relationships with clinical team. In particular listen for issues around perceived benefit of reviews and relationships with healthcare professionals.	
Tell me about your healthcare team?<br>- Do you go to all your appointments about your diabetes?<br>- If no: Help me understand why not? (then move on to next block)	
What are the best and worse bits about going to clinic appointments?	
What advice would you give to another diabetic about their appointments?	
Do you feel there are things about living with diabetes that don't get discussed at clinic appointments? Tell me about that? What would it mean if you were able to discuss these?	

Section two (2.5) – diabetes in general	
Information about this section (NOT for discussion with participant):<br><br>This section is to give the participant opportunity to share ideas and priorities. In particular listen for things they would like to change that would help them feel confident to live well.	
Overall, how important is diabetes management in your life right now?	10mins
How do you prioritise diabetes compared to some other things in life?	
What would you say are the top 3 things that this work should focus on to help young people with diabetes to live well and stay positive?<br>- If needed, could say "for example, would you like to practice how you introduce your diabetes into a conversation with a new friend or partner"	

University of Brighton protocol 2022-9871
For use in this study only

Close

Before we finish the call today, are there any other comments you'd like to make or things you would like us to consider that we haven't spoken about.

We've come to the end of time today. I've learned a lot about your experiences. Thank you for sharing your thoughts and stories with me.

We are also trying to find some brothers/sisters/friends/parents/carers/teachers of people with diabetes and I wonder if you have a sibling or friend or partner or teacher or if one of your parents/carers might be willing to talk to me as well. I would not share any information about our conversation today – everything you said stays completely confidential. You would be welcome to attend as well if you prefer, or I could just speak to them on their own. Is there anyone who you would like to recommend for this?

- [If yes: capture name and provide info for sibling/significant other]
- [If no: No problem. If you change your mind, please feel free to drop me a message]

As a thank you for your time today, I can send you a £20 amazon voucher either in the post, or via email. How would you like me to send it to you?

- [If likely to speak up in a group:]

In the next phase, we will be conducting a small focus group discussion with young people with diabetes to look at a prototype toolkit and get some feedback. We will also be designing a research study together in the group. Would you be interested to participate in this group? I don't have the dates yet but can confirm nearer to time to see if you are availability if you would like to participate.

- [If yes: Is it ok to keep your name and contact details so I can contact you about this?][confirm via email immediately after interview, and include an “unsubscribe” option in case they change their mind]
- [If no: No problem. I just thought I would ask.]

Would you like me to send you a summary of what we learn from all the interviews like this that we are doing?

- [If yes: Is it ok to keep your name and contact details so I can contact you about this?][confirm via email immediately after interview, and include an “unsubscribe” option in case they change their mind]
- [If no: No problem. I just thought I would ask.]

University of Brighton protocol 2022-9871
For use in this study only

ISSUE MANAGEMENT

Information about this section (NOT for discussion with participant):

Use this Section if required to deal with issues that may come up during the discussion.

If participant's temperament or comprehension changes during the interview, say:

- Would you like a break? Maybe you'd like to check your blood sugar.
- I can stay on the call, or give you a call back in 15 minutes.

If participant becomes upset whilst relaying experiences, say:

- I can see this is upsetting for you, and I understand it is hard to talk about. Thank you so much for being so open with me. What you have told me is really helpful for this research.
- Are you happy to carry or would you like a 5 minute break. I can call you back.

If participant discloses issues that may affect their physical or mental health (including but not limited to insulin avoidance, self-harm, use of drugs, bullying, etc), say:

- Thank you for being so open with me today. You've told me some things that have worried me about [restate issue using participant's language]. I really encourage you to speak to someone in your healthcare team about this. Can you do that for me?
- Refer to Participant Information Sheet
- Follow up email/message to include appropriate links to patient groups from the following list and will encourage them to speak to their healthcare team.
  - Diabetes UK
  - Diabetics with eating disorders
  - T1 Teens Instagram group
  - YoungMinds
  - The samaritans

If participant discloses suicidal thoughts, any safeguarding issues, or anything life threatening or that may impact their immediate safety, say:

- Thank you for being brave enough to tell me that. I'm really concerned about this and would like to make sure you get some help as quickly as possible. Is there someone you can talk to today about this? I would like you to tell them what you've told me, which I've noted as "[restate using participant's language]"
- Would you be ok for me to contact someone for you? Who would you prefer me to call?

- Follow up email/message to include appropriate links to patient groups from the following list and will encourage them to speak to their healthcare team
  - The samaritans
  - Childline

Additional steps may be required depending on the severity of the reported issue. To be determined on a case-by-case basis in conjunction with supervisors, university and PIs if appropriate. Anonymity will be protected wherever possible.

University of Brighton protocol 2022-9871
For use in this study only

Interview guide #2

1:1 research interview with SIBLINGS/PEERS of Young Persons with Type 1 Diabetes (part 1 of study)

This checklist is a reminder to the researcher to cover specific areas within the interview. Below the checklist is the interview guide that will be used to guide the conversation

CHECKLIST	
Introduction	
Introduce self by name	
Qualify what name they would like to be used in the interview	Name (first name/nickname only):
Confirm they have had the participant information sheet – share / read through if not	
Advise on anticipated duration of call	
Confirm agreement to recording the interview and explain data handling	
Advise not to use people’s full names. Suggest they use terms like “doctor” or “teacher” or “sister” or “brother” or initials instead of stating their name	
Explain the topics of the discussion today	
Explain what the information will be used towards	
Ensure informed consent is captured	
Discussion	
Cover each topic as indicated below	Note here any topics not covered, and reason why:
• Setting the scene	
• Diabetes and life	
• Diabetes and family	
• Diabetes and friends/school/work/significant other	
• Diabetes in general	
Interview close	
Check for any outstanding comments or reflections	
Thank the participant	
Explain reimbursement and confirm details	
Confirm if they would like to receive the final report about the research once we have developed and tested the intervention	

University of Brighton protocol 2022-9871
For use in this study only

Introduction

> Information about this section (NOT for discussion with participant):
>
> This section is to build rapport, ensure the participant understands about the research interview, confidentiality and consents to participating.

Thank you for meeting with me today to talk about your experiences of [living with/being friends with] someone with type 1 diabetes.

My name is [insert name of researcher]. What would you like me to call you as we chat? Please just use a first name or a nickname.

Can I just check that you got the participant information sheet from me or your [brother/sister/friend]?

Before we start, let me explain what our call is about today. I'm a researcher, here to learn about how diabetes fits into the lives of young people. I'm keen to learn your thoughts about what your [brother/sister/friend] finds most difficult about living day to day with diabetes. What you share will help us to design a toolkit that seeks to help people in their late teens/early twenties to build their lives around diabetes.

As we discuss this, I would welcome your honest thoughts and feedback – not what you think I might want to hear. There are no right or wrong answers. I'm not here to tell you what you should/shouldn't be doing or how you should/shouldn't be feeling. It's your personal story that I'm interested in.

Everything you say is completely private. That's why we are just going to use first names – it means that no-one else will know that it was you who said any of the things you tell me. The only time I would need to speak to someone else is if I was concerned for your (or another person's) immediate safety. I would normally ask you if that's ok and you could tell me who you would prefer me to speak to.

University of Brighton [illegible] 2022-9871
For use in this study only

Our chat will last about 30 minutes. If you need a break at any point please just say. We can easily take a break if needed.

Are you happy to continue with this research talk today?

- [If timing is an issue – agree appropriate next steps with participant eg rescheduling to a more convenient time]

Before we start I need to make sure that you understand what we are using the information you give me for and that you are agreeing freely to participate. This is called "informed consent". Have you had the informed consent sheet?

- [If so – check you have evidence, and reconfirm they are happy to proceed.
    - "Thanks for completing the form for me. Can I just confirm you are still happy to proceed today?"]
- [If not – read through Informed Consent sheet and ensure verbal agreement to proceed. Send IC form via email afterwards]
    - "No problem. I do need to make sure I have your informed consent though so let me read through the points with you and you can tell me if you agree with each of them. I will send you the form afterwards for your records."

- [If informed consent is not obtained, do not proceed]
    - "That's absolutely fine. You are free to decide whether or not to take part and I respect your decision not to participate. If you change your mind in the future, please feel free to send me a message but for now I understand that you don't want to participate. I wish you all the best for the rest of the day."

University of Brighton protocol 2022 9871
For use in this study only

If you agree, I would like to record our talk so I can make sure to write good notes and really understand your experiences. The only people who will listen to the recording are me, and two other researchers in my team.

Are you happy for me to record our conversation?

- [If yes – start recording]
- [If no - check what time they have available. Explain 'we will likely need a little more time for our chat. Is that ok today? It's just that I need to write notes as we talk.]

State interview number and date for the recording

Diabetes discussion

Section one – setting the scene	
Information about this section (NOT for discussion with participant):<br>This section is to build further rapport, and give the participant a chance to open up and start talking. Day to day management will be used as a mapping question linked to age and duration of T1D	
Please tell me a little bit about yourself? How old are you, who you live with, what you do, etc?	5 mins
How long have you known about your [brother/sister/friend]'s diabetes?	

Do you ever have to do anything to help them manage their diabetes? If so, what	

Section two (2.1)– diabetes and life	
Information about this section (NOT for discussion with participant):<br><br>This section is to understand general insights and challenges about living with diabetes. In particular listen for perceived limitations or difficulties	
From what you see with your [brother/sister/friend], how would you describe living with diabetes day to day?<br>- What, if anything do you think makes it hard to live with diabetes?<br>- What words would you use to describe diabetes?	10 mins
Sometimes there are specific times in life that really stick in our minds. Are there any specific examples you remember where diabetes made it hard for your [brother/sister/friend] to do something?<br>- Tell me about that.<br><br>Alternative Q (use if they struggle with above Q): What has been the hardest thing in the last week for your [brother/sister/friend] about their diabetes?<br>- Could anything have make it easier or more difficult	
How do you think they cope with diabetes day to day?<br>- Tell me about those thoughts?	
Are there any things you think they don't cope well with, or could cope better with?	
What advice would you like to give them about living with diabetes, that maybe you've never said to them?	

University of Brighton protocol 2022-9871
For use in this study only

Section two (2.2) – FOR BROTHERS/SISTERS ONLY diabetes and family	
Information about this section (NOT for discussion with participant):<br><br>This section is to understand insights and challenges about family life for a diabetic. In particular listen for causes of family conflict and causes of any relationship difficulties .	
How does diabetes fit into your family life?<br>- (If needed, can say "Do family help your [brother/sister] with their diabetes at all, or they tell you what they think you should do?)<br>- (If needed, can say "Have there been times when diabetes has caused difficulties or arguments with any family members?<br>o Tell me about that.)	10 mins

How would your [brother/sister] would cope with their diabetes if all the family weren't there for a week?<br>- How do you think they would feel about that?	

Section two (2.3) – FOR FRIENDS/PEERS ONLY diabetes and school/college/university/work and friends/significant other	
Information about this section (NOT for discussion with participant):<br><br>This section is to understand relationships with peers and/or partners. In particular listen for issues around disclosure, comments from peers, avoidance strategies to managing diabetes.	
Tell me about how diabetes fits into your friend's life at school/college/university/work?<br>- Are there any times when it's hard for [him/her] to deal with diabetes at school/work?	10mins
What would be the best way for them to deal with diabetes at school/college/university/work?	
Do they tell other friends/significant other(s) that they have diabetes?	
What do you think they find difficult about diabetes in school/college/university/work?	
Is there anything *you* find challenging about their diabetes?	

University of Brighton protocol 2022-
r use in this study only

Section two (2.4) – diabetes in general	
Information about this section (NOT for discussion with participant):<br><br>This section is to give the participant opportunity to share ideas and priorities. In particular listen for things they would like to change that would help YPT1D feel confident to live well.	
Overall, how important do you think managing diabetes tasks is in your [brother/sister/friend]'s life right now?	10mins
How do they prioritise diabetes compared to some other things in life?	
What would you say are the top 3 things that this work should focus on to help young people with diabetes to live well and stay positive?<br>- If needed, could say "for example, how to introduce your diabetes into a conversation with a new friend or partner"	

Close

Before we finish the call today, are there any other comments you'd like to make or things you would like us to consider that we haven't spoken about.

We've come to the end of time today. I've learned a lot about your experiences. Thank you for sharing your thoughts and stories with me.

As a thank you for your time today, I can send you a £20 amazon voucher either in the post, or via email. How would you like me to send it to you?

ISSUE MANAGEMENT

Information about this section (NOT for discussion with participant):

Use this Section if required to deal with issues that may come up during the discussion.

If participant becomes upset whilst relaying experiences, say:

- I can see this is upsetting for you, and I understand it is hard to talk about. Thank you so much for being so open with me. What you have told me is really helpful for this research.
- Are you happy to carry or would you like a 5 minute break. I can call you back.

If participant discloses issues that may affect their physical or mental health (including but not limited to self-harm, use of drugs, bullying, etc), say:

- Thank you for being so open with me today. You've told me some things that have worried me about [restate issue using participant's language]. I really encourage you to speak to someone about this. Can you do that for me?
- Ask who they would speak to and recommend a doctor, parent, teacher depending on their personal situation.
- Follow up email/message to include appropriate links to patient groups from the following list and will encourage them to speak to their doctor.
    - YoungMinds
    - The samaritans

If participant discloses suicidal thoughts, any safeguarding issues, or anything life threatening or that may impact their immediate safety, say:

- Thank you for being brave enough to tell me that. I'm really concerned about this and would like to make sure you get some help as quickly as possible. Is there someone you can talk to today about this? I would like you to tell them what you've told me, which I've noted as "[restate using participant's language]"
- Ask who they would speak to and recommend a doctor, parent, teacher depending on their personal situation.
- Would you be ok for me to contact someone for you? Who would you prefer me to call?
- Follow up email/message to include appropriate links to patient groups from the following list will encourage them to speak to their doctor

University of Brighton prot[illegible]2-98[illegible]1
For use in this study only

- The samaritans
- Childline

Additional steps may be required depending on the severity of the reported issue. To be determined on a case-by-case basis in conjunction with supervisors, University and PIs if appropriate. Anonymity will be protected wherever possible.

University of Brighton protocol 2022-9871
For use in this study only

Facilitator Guide

Focus Group Discussion with YPT1D (part 2 of study)

This information will be used to shape the focus group with young people with type 1 diabetes. The detailed of the workshop guide will be refined or rebuilt following insights from phase 1a interviews.

Examples of provided in the tables as a starting point to illustrate intent.

CHECKLIST	
Introduction	
Confirm each participant has given informed consent on entry. Ensure this is documented	
Confirm they have had the participant information sheet	
Introduce self by name	
Round table introductions – first names only	
Confirm agreement to recording the discussion and explain data handling	
Advise not to use people's full names. Suggest they use terms like "my doctor" or "my teacher" or initials instead of staying their name	
Explain the main themes of the discussion today	
Explain what the information will be used towards	
Discussion	
Agree ways of working for workshop	
Cover each topic as indicated below	
Focus group close	
Check for any outstanding comments or reflections	
Thank the participants	
Explain reimbursement and confirm details	
Confirm if they would like to receive the final report about the research once we have developed and tested the intervention	

University of Brighton protocol 2022-9871
For use in this study only

Capturing informed consent

On entry, prior to meeting start:

Before we start I need to make sure that you understand what we are using the information you give me for and that you are agreeing freely to participate. This is called "informed consent". Have you had the informed consent sheet?

- [If so – check you have evidence, and reconfirm they are happy to proceed.
  - "Thanks for completing the form for me. Can I just confirm you are still happy to proceed today?"]

- [If not – provide if face-to-face and ensure completed by participant. If videocall, read through Informed Consent sheet and ensure verbal agreement to proceed. Send IC form via email afterwards]
    - eg "No problem. I do need to make sure I have your informed consent though so let me read through the points with you and you can tell me if you agree with each of them. I will send you the form afterwards for your records."

- [If informed consent is not obtained, do not proceed]
    - "That's absolutely fine. You are free to decide whether or not to take part and I respect your decision not to participate. If you change your mind in the future, please feel free to send me a message but for now I understand that you don't want to participate. I wish you all the best for the rest of the day."

I will be recording our workshop so I can make sure to write good notes and really understand everyone's input. The only people who will listen to the recording are me, and two other researchers in my team.

University of Brighton protocol 2022-9871
For use in this study only

Also, let me check I have the correct contact details for you so I can send you your Amazon voucher after the workshop.

- Confirm email, or address if they want a voucher sent in the post. Store confidentially including during workshop

<u>Introduction</u>

Thank you all for meeting with me today to discuss how we design a toolkit for young people with type 1 diabetes to use in day-to-day life to help them live well with diabetes.

Can I just check that you all got the participant information sheet from me or your nurse?

As a reminder, I will be recording our workshop so I can make sure to write good notes and really understand everyone's input. The only people who will listen to the recording are me, and two other researchers in my team. I'm just going to start the recording now.

My name is [insert name of facilitator. I'm looking forward to hearing your thoughts and ideas so can we just go round the room and say hello and tell each other our name. Please just use a first name or a nickname.

Before we start, let me explain what our workshop is about today. I'm a researcher, designing a toolkit for young adults with type 1 diabetes. I think it is best suited to people who are between 16 and 21 years old, but you might tell me otherwise today.

As we discuss this, I would welcome your honest thoughts and feedback – not what you think I might want to hear. There are no right or wrong answers. I'm not here to tell you what you should/shouldn't be doing or how you should/shouldn't be feeling.

Please treat all information in this workshop as private. Some people might share personal stories or opinions and it's important that we each respect their opinion and their privacy. That's why we are just going to use first names and we're not going to talk about specific doctors, hospitals, schools, colleges, etc. The only time I would need to speak to someone else is if I was concerned for your (or another person's) immediate safety. I would ask you if that's ok and you could tell me who you would prefer me to speak to.

Our workshop will last about 3 hours. We have a break scheduled halfway through but if you need a break, or a snack, at any point please just say. We can easily take additional short breaks if needed.

Before we start, I'd like us to agree how we want to work together in this workshop. I've put together these points to start us off. Do you agree and/or is there anything else you would like to add or change here?

- Show Ways of Working in our Workshop slide (see Figure 1 Ways of Working in our Workshop Slide)
- Annotate slide with suggestions from participants.
- Leave this document on display during the session

protocol 2022-9871

Figure 1 Ways of Working in our Workshop Slide

Ways of Working in our Workshop

University of Brighton

What are we here to do today?

- Discuss your ideas and opinions about a toolkit that is being designed for young people with type 1 diabetes to use in day-to-day life to help them live well with diabetes.
- Together we will (a) design the toolkit and (b) discuss how we will measure if it is helpful and easy to use

How do we want to work together today?

- Respect the privacy of this meeting – what is said in the room, stays in the room. We won't talk about what other people say outside this meeting
- Give everyone an opportunity to speak and share ideas
- Respect each others ideas and opinions, even when different to our own
- Recognise that we have different experiences and each of us might have very different lives (at home, at school/college/work, etc)
- Be honest
- Be creative
- Have fun

Version SSHS PHD V1.0 Jan 2022

Part One – Agree what an intervention to help young people to live well with Type 1 Diabetes should address

The following tables follow the principles based on the Behavioural Change Wheel Guide to Designing Interventions [1]. The tables below are derived from this guide and workshops will be designed following the guidance given by Michie *et al* in this guide.

Defining the problem in behavioural terms

This table is based on PPI observations and will be refined following phase 1a interviews

What behaviour am I seeking to change?	Improving self-efficacy and coping strategies for young adults aged 16-21 years living with type 1 diabetes (target population) and enable them to effectively manage diabetes-related tasks. Improve resilience and optimism amongst the target population
Where does this behaviour occur?	School/college/university, place of work and social situations with peers
Who is involved in performing this behaviour?	The young person with diabetes<br>Their teacher(s)<br>Their parent(s)/carer(s)<br>Their manager/colleagues<br>Their peers<br>Their partner

University of Brighton protocol 2022-9871
For use in this study only

Candidate target behaviours that could improve the targeted behaviour of self-efficacy and coping strategies.

This table is built from PPI insights and observations from grey literature. It will be refined following phase 1a interviews

Observe role models with type 1 diabetes – celebrities/well-known individuals/characters.<br>Connect with buddy with type 1 diabetes<br>Connect with peer group for support<br><br>Build a vision for the future (Have a goal – what are the steps to get to the goal?)<br><br>See Value in planning and managing diabetes (what does it give me, what does it avoid)<br><br>Build Confidence to "do" tasks (eg assess insulin requirements, dose adjust insulin, plan for activity)<br>Build Confidence to "deal with" issue (eg hypo/hyper – what words and what actons)<br>Build Confidence to "speak up" (eg language to explain diabetes related tasks, asking for help)<br>Build Confidence in "choices" (eg sex, alcohol, food, drugs)(eg holidays)<br>Build Confidence in HCP network (eg ok to be honest, ask for help, seek opinion without judgement)

Recognising the importance of a plan (for activities, for day-to-day, for the unexpected)<br>Ability to make a plan (eg have food/insulin available, how to get help if needed)<br>Confidence in the personalised plan<br><br>Acceptance that sometimes things will go wrong – how to get back on track, how to avoid self-blame

University of Brighton protocol 2022-9871
For use in this study only

Selecting target behaviour for this intervention

This table presents a summary of ideas for potential target behaviours.

This table will be redefined during phase 1b workshops with YPT1D and carers

Potential target behaviour	Impact of behaviour change	Likelihood of behaviour change	Spillover score	Measurement score
Observe role models with type 1 diabetes – celebrities/well-known individuals/characters.	2	1	2	1
Connect with buddy with type 1 diabetes	2	2	2	3
Connect with peer group for support	3	3	3	3
Build a vision for the future (Have a goal – what are the steps to get to the goal?)	2	2	3	4
See Value in planning and managing diabetes (what does it give me, what does it avoid)	3	2	3	2
Build Confidence to "do" tasks (eg assess insulin requirements, dose adjust insulin, plan for activity)	4	4	3	4
Build Confidence to "deal with" issues (eg hypo/hyper – what words and what actons)	4	4	4	3
Build Confidence to "speak up" (eg language to explain diabetes related tasks, asking for help)	4	3	3	4
Build Confidence in "choices" (eg sex, alcohol, food, drugs)(eg holidays)	4	2	2	3
Build Confidence in HCP network (eg ok to be honest, ask for help, seek opinion without judgement)	3	3	4	4
Recognising the importance of a plan (for activities, for day-to-day, for the unexpected)	4	3	3	4
Ability to make a plan (eg have food/insulin available, how to get help if needed)	4	3	3	4
Confidence in the personalised plan	4	3	3	4
Acceptance that sometimes things will go wrong – how to get back on track, how to avoid self-blame	4	2	3	4

1= unacceptable, 2 = unpromising by worth considering, 3 = promising, 4 = very promising

HIGHLIGHTS anticipated selected target behaviours

[illegible]iversity of Brighton protocol 2022-9871
[illegible]se in this study only

Specifying the target behaviour

This table will be redefined during phase 1b workshops with (i) YPT1D and (ii) carers

Target behaviour	Confidence and capability to live independently with diabetes
Who needs to perform the behaviour?	The young person with T1D
What do they need to do differently?	• Build Confidence to "do" tasks (eg assess insulin requirements, dose adjust insulin, plan for activity)<br>• Build Confidence to "deal with" issues (eg hypo/hyper – what words and what actons)<br>• Build Confidence to "speak up" (eg language to explain diabetes related tasks, asking for help)<br>• Build Confidence in HCP network (eg ok to be honest, ask for help, seek opinion without judgement)<br>• Recognising the importance of a plan (for activities, for day-to-day, for the unexpected)<br>• Ability to make a plan (eg have food/insulin available, how to get help if needed)<br>• Confidence in the personalised plan
When do they need to do it?	When they reach independence (leave school/home), or within 6 months of starting intervention
Where do they need to do it?	At school/college/university<br>At work<br>When socialising with friends
How often do they need to do it?	Every day
With whom do they need to do it?	The young person with diabetes<br>Their parent(s)/carer(s)<br>Their teacher(s)<br>Their manager/colleagues<br>Their peers<br>Their partner

University of Brighton protocol 2022-9871
For use in this study only

Identifying what needs to change

This table will be redefined during/after phase 1b workshops with (i) YPT1D and (ii) carers

COM-B components	What needs to happen for the target behaviour to occur?	Is there a need for change?
Physical capability	Know how to treat high and low blood sugar<br>Be able to take blood readings	No – this should be covered by clinical education and family support

	Be able to self inject or use pump<br>Be able to count carbs<br>Be able to dose adjust	These are required prior to entry into this programme
Psychological capability	Understand the role of insulin and food on blood sugar<br>Understand what is a hypo, what is hyper and what is DKA<br><br>Understand influence of activities on diabetes<br>Understand influence of stress on diabetes	Partly yes – effect of activities and stress can vary person to person so may need further development beyond what is gained from clinical education<br>Learning the biology of diabetes will be covered elsewhere (eg clinical education)
Physical opportunity	Needing to manage diabetes self-management tasks in a variety of situations including with peers, colleagues, teacher<br>Needing to ask to help to treat a hypo<br>Needing to make a plan and change a plan which managing diabetes effectively (eg walking home from school instead of taking the bus, invite to a party at short notice)	Yes
Social opportunity	Have a degree of control over food choice(s)<br>Be able to control choice of activity(ies)<br>Having social and/or work network of peers/colleagues<br>Meeting new people who need to know about diabetes	Maybe – although anticipate this will happen automatically as life changes for the young person
Reflective motivation	Desire to be independent, and able to live a normal life	This is a great goal and achievable aspiration.<br>Some participants may need confidence building that they will be able to achieve this, but otherwise it is anticipated to be a natural desire of young people
Automatic motivation	Feeling comfortable with living confidently with diabetes in day to day life	Yes.

University of Brighton protocol 20229871
For use in this study only

	Feeling confident to deal with diabetic related issues	

Part Two – Reviewing the Prototype Intervention to help young people to live well with Type 1 Diabetes should address

A prototype intervention will be shared with the participants who will be asked to review whether it meets the areas of focus outlined above.

Questions will be addressed.

Part Three – Adapting the Prototype Intervention to help young people to live well with Type 1 Diabetes should address

The group will be asked to annotate and suggest edits that, in their individual opinion, would make the intervention valuable for, and usable by, young people with diabetes.

Part Four – Measuring the Effectiveness of an Intervention to help young people to live well with Type 1 Diabetes should address

The prototype design of a pilot study and the proposed outcomes will be shared with the group and the group will be asked to reflect on and make recommendations if the questionnaire based outcomes are appropriate and whether they would like to consider other outcomes within the study.

University of Brighton protocol 2022-987
For use in this study only

1. Michie S., A.L., West R, *Behaviour Change Wheel - A Guide to Designing Interventions*. 2014, Great Britain: Silverback Publishing.

ISSUE MANAGEMENT WITHIN FOCUS GROUP DISCUSSION

Information about this section (NOT for discussion with participant):

Use this Section if required to deal with issues that may come up during the discussion.

If a participant's temperament or comprehension changes during the interview, speak privately to them and say:

- Would you like a break? Maybe you'd like to check your blood sugar.
- You can step out if you need to.

If participant becomes upset whilst relaying experiences, say:

- I can see this is upsetting for you, and I understand it is hard to talk about. Thank you so much for being so open with me. What you have told me is really helpful for this research.
- Are you happy to carry or would you like a 5 minute break. You can step out if you need to

---

If participant discloses issues that may affect their physical or mental health (including but not limited to insulin avoidance, self-harm, use of drugs, bullying, etc), say:

- Thank you for being so open with me today. You've told me some things that have worried me about [restate issue using participant's language]. I really encourage you to speak to someone in your healthcare team about this. Can you do that for me?
- Refer to Participant Information Sheet
- Follow up email/message to include appropriate links to patient groups from the following list and will encourage them to speak to their healthcare team.
    - Diabetes UK
    - Diabetics with eating disorders
    - T1 Teens Instagram group
    - YoungMinds
    - The samaritans

If participant discloses suicidal thoughts, any safeguarding issues, or anything life threatening or that may impact their immediate safety, say:

- Thank you for being brave enough to tell me that. I'm really concerned about this and would like to make sure you get some help as quickly as possible. Is there someone you can talk to today about this? I would like you to tell them what you've told me, which I've noted as "[restate using participant's language]"
- Would you be ok for me to contact someone for you? Who would you prefer me to call?

- Follow up email/message to include appropriate links to patient groups from the following list and will encourage them to speak to their healthcare team
    - The samaritans
    - Childline

Additional steps may be required depending on the severity of the reported issue. To be determined on a case-by-case basis in conjunction with supervisors, university and PIs if appropriate. Anonymity will be protected wherever possible.

If dispute or argument breaks out between participants, say:

University of Brighton protocol 2022-9871
For use in this study only

- Everyone has different perspectives, and each of us has the right to share our opinion. We don't have to agree on everything.
- What you are each saying is important and I want to make sure we capture the different opinions you are sharing. Why don't you write them down for me or record a short voice recording on your phone if you prefer and give it to me before the end of the workshop today. You are welcome to contact me after the workshop too (provide contact details).

University of Brighton protocol 2022-9871
For use in this study only
